# Application of Antioxidant Compounds in Bone Defect Repair

**DOI:** 10.3390/antiox13070789

**Published:** 2024-06-28

**Authors:** Jiajia Wang, Yubing Zhang, Qingming Tang, Yinan Zhang, Ying Yin, Lili Chen

**Affiliations:** 1 Department of Stomatology, Union Hospital, Tongji Medical College, Huazhong University of Science and Technology, Wuhan 430022, China; 2 School of Stomatology, Tongji Medical College, Huazhong University of Science and Technology, Wuhan 430030, China; 3 Hubei Province Key Laboratory of Oral and Maxillofacial Development and Regeneration, Wuhan 430022, China; 4 School of Chemical Science and Engineering, Tongji University, Shanghai 200092, China

**Keywords:** bone defect, antioxidant compounds, biomaterials, ROS

## Abstract

Bone defects caused by trauma, tumor resection, and infections are significant clinical challenges. Excessive reactive oxygen species (ROS) usually accumulate in the defect area, which may impair the function of cells involved in bone formation, posing a serious challenge for bone repair. Due to the potent ROS scavenging ability, as well as potential anti-inflammatory and immunomodulatory activities, antioxidants play an indispensable role in the maintenance and protection of bone health and have gained increasing attention in recent years. This narrative review aims to give an overview of the main research directions on the application of antioxidant compounds in bone defect repair over the past decade. In addition, the positive effects of various antioxidants and their biomaterial delivery systems in bone repair are summarized to provide new insights for exploring antioxidant-based strategies for bone defect repair.

## 1. Introduction

Bone defects are common and serious clinical problems for orthopedists. The damage inflicted by bone defects primarily impacts the patient’s mobility, and in severe cases, it may even lead to limb deformities and loss of function. These defects not only diminish individual quality of life, but also impose a substantial economic burden on public health systems, with annual healthcare costs reaching billions of dollars for treatments related to bone defects [1].

There are various causes of bone defects, such as comminuted fractures; open fractures; and large bone tissue defects due to trauma, osteonecrosis, and bone separation as a result of systemic inflammation, as well as bone defects caused by tumor resection or surgery. It is necessary to increase the amount of new bone by promoting the osteogenic activity at the site of a bone defect to achieve the purpose of treating bone defects.

The complex process of bone defect healing encompasses cellular proliferation and differentiation, inflammatory responses, and management of oxidative stress. The spontaneous healing capacity of bone defects depends on the defect’s size, with minor defects potentially being able to heal independently. However, when defects surpass a critical size, they require medical intervention to facilitate and expedite the healing process. This is due to challenges such as inadequate blood supply and local infections, which hinder natural regeneration [2]. Numerous studies have been dedicated to exploring more effective treatments for large bone defects [3].

Free radicals, encompassing reactive oxygen species (ROS) and reactive nitrogen species (RNS) like hydrogen peroxide (H_2_O_2_), superoxide ions (O_2_-, hydroxyl radical (OH-), and nitrogen oxide (NO), arise from routine cellular activities as well as external environmental factors such as mitochondria, ischemia, or radiation exposure [4,5]. While these radicals are essential for bactericidal activities and exert influence over cellular proliferation and differentiation, their excessive production or insufficient elimination can induce oxidative stress (OS). This disrupts various physiological processes and contributes to the pathogenesis of various diseases, including cancer, cardiovascular diseases, and skeletal system disorders [5,6,7]. During bone defect repair, osteoblasts, osteoclasts, and osteocytes interact in a coordinated manner with various growth factors and cytokines to maintain continuous bone formation. Abnormalities in redox status disrupt this bone regeneration process, while ROS activate differentiation of pre-osteoclasts as well as induce apoptosis of osteoblasts and osteocytes [8]. In addition, ROS alter the receptor activator κB ligand (RANKL)/osteoprotegerin (OPG) ratio through signaling pathways such as extracellular signal-regulated kinases (ERK1/2) and c-Jun-N terminal kinase (JNK) [9,10,11]. These cellular and molecular alterations lead to an imbalance between osteogenic and osteoclastic activities in the defect area, ultimately delaying the process of bone repair.

Scavenging free radicals is important for promoting bone repair, which requires antioxidants to function. Common antioxidants, such as polyphenols, astaxanthin, α-lipoic acid, vitamin E, and flavonoids, have undergone extensive study for their therapeutic potential [12,13,14,15,16]. These compounds can be administered through various routes, including oral ingestion, injection, or incorporation into implantable devices, to address a multitude of conditions, such as cartilage and bone repair, by restoring the equilibrium between ROS production and elimination [17,18,19,20]. Indeed, achieving the delicate balance between mitigating harmful ROS as mediators of tissue damage and preserving their advantageous functions, such as antimicrobial properties, presents a significant challenge. This underscores the need for further research to unravel the intricate dynamics between antioxidants and ROS in therapeutic interventions.

While existing reviews have touched on the role of inhibiting oxidative stress in bone repair, their discussion has focused mainly on the role of biomaterials in ROS [7,21]. This review summarizes the application of antioxidant compounds in bone repair therapy over the past decade to elucidate various approaches for delivering antioxidant compounds to the healing sites of bone defects and describing their respective advantages and disadvantages. Our perspective is intended to guide the future research direction for clinical application and pave the way for the study of more effective and targeted antioxidant treatment strategies for bone defects.

## 2. Methods

**Figure 1:**
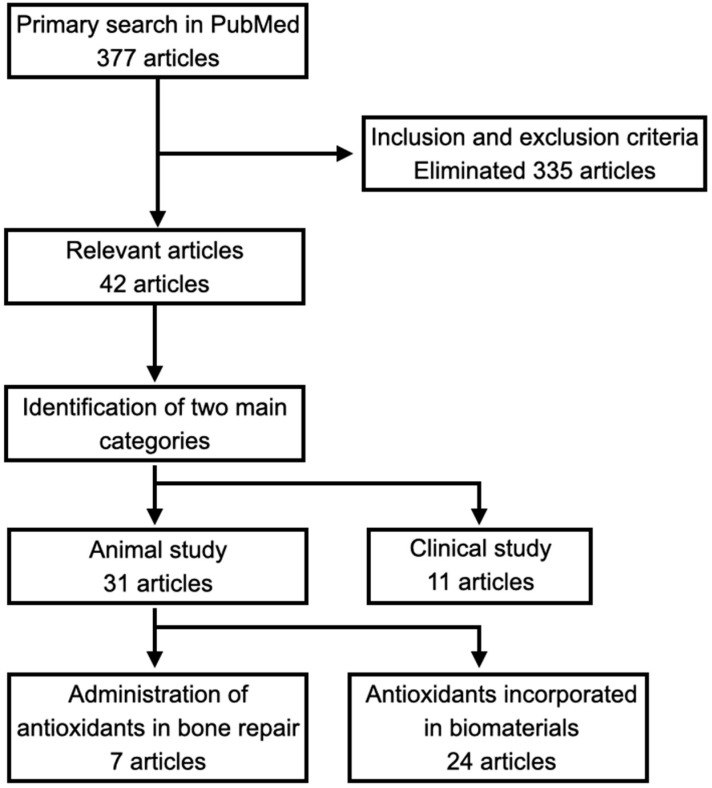
The literature search and selection process.

To identify articles on the application of antioxidant compounds in bone defect repair, a literature search was performed in PubMed using various combinations of a set of keywords: “Reactive Oxygen Species”, “Antioxidants”, “Antioxidant Compounds”, “Bone Defect”, and “Bone Repair”. English articles published between 2014–2024 were selected. In addition, selected articles published before 2014 on the role of ROS/antioxidants in the human skeletal system were included. The initial search and screening retrieved 377 articles. Articles that did not involve in vivo models or antioxidant compounds, or which were non-original research articles, were excluded. Finally, 42 articles were included (Figure 1). The inclusion of articles on the effects and mechanisms of ROS on bone repair was not strictly restricted by the type of article or time of publication.

## 3. ROS and Oxidative Stress in Bone Repair Process

The most critical substance for oxidative stress is ROS, so most of the literature on promoting bone loss repair addresses oxidative stress impediments to bone healing by removing ROS [7]. ROS are predominantly generated within mitochondria as by-products of energy metabolism [22]. When maintained at controlled levels, ROS play a crucial role in normal cellular signaling mechanisms involved in bone regeneration, significantly influencing cellular communication and regulating fundamental cellular processes such as proliferation, differentiation, apoptosis, repair processes, and inflammation [23,24]. Through their involvement in cell signaling, ROS contribute to orchestrating activities between different cell types within the bone microenvironment, facilitating communication and cooperation necessary for effective bone repair and regeneration [25]. This delicate balance is maintained by a complex endogenous antioxidant system composed of enzymatic and non-enzymatic proteins capable of neutralizing reactive species or ensuring low production. However, when this balance is disrupted due to excessive ROS production or insufficient antioxidant defenses; for example, in an inflammatory state, OS occurs, potentially impacting bone repair [26,27].

**Figure 2:**
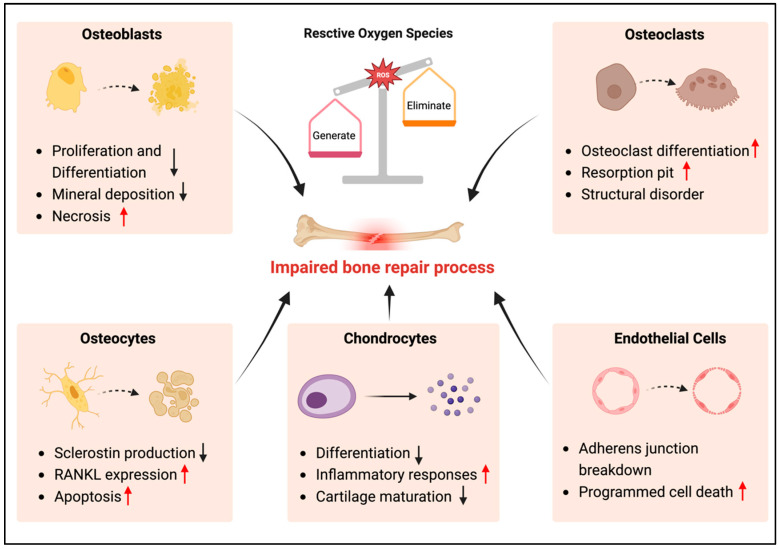
Effects of ROS on the major cells involved in bone repair. Top left panel: Excess ROS inhibit osteoblast proliferation and differentiation, leading to deficient mineral deposition. Top right panel: Excess ROS promote osteoclast differentiation, leading to increased resorption pits and bone structural disorder. Bottom left panel: Excess ROS inhibit sclerostin production by osteocytes and promote RANKL expression. Bottom middle panel: Excess ROS inhibit chondrocyte differentiation and enhance cellular inflammatory response, leading to impaired cartilage maturation. Bottom right panel: ROS cause adherens junction breakdown and cell death of endothelial cells. The arrows in the figure indicate increasing (red) or decreasing (black). Created with BioRender.com (accessed on 17 June 2024).

Bone repair is a dynamic and complex biological process involving multiple cell types and molecular signals, and is primarily achieved through intramembranous and endochondral ossification mechanisms [28]. This reparative journey unfolds across four distinct phases: the inflammation phase, cartilage callus phase, hard bone callus phase, and remodeling phase. Excess ROS affect the process of bone repair by altering the biological function of the major cells involved in bone remodeling (Figure 2).

### 3.1. Inflammation Phase

The inflammation phase lays the groundwork for subsequent healing phases and bone regeneration. The inflammation phase commences with hematoma formation resulting from blood vessel rupture, followed by a cascade of cytokine and growth factor release aimed at attracting inflammatory cells for debris clearance [29,30]. Among the inflammatory cells recruited to the site are neutrophils, macrophages, and lymphocytes, pivotal in phagocytosing cell debris and pathogens [31]. These phagocytes use NADPH oxidase to produce high levels of ROS as part of their microbiome-killing mechanism [32]. However, the overproduction of ROS can react against these immune cells. Upregulated ROS may impair the initiation and outcome of phagocytosis, leading to oxidative bursts and the production of neutrophil extracellular traps [33,34,35]. In addition, ROS can reduce neutrophil migration by impairing CXCR2 function [36]. As for macrophages, Zhang et al. reported that ROS clearance and inhibition block the differentiation of M2 macrophages, which promote osteogenic differentiation [37,38]. Similarly, it has been reported that excessive ROS affects the polarization, activation, and function of lymphocytes [39].

Endothelial cells lining the blood vessels also play crucial roles during the inflammatory phase [40]. Endothelial cells work to re-establish the vascular network within the healing bone, essential for delivering nutrients, oxygen, and immune cells to the site. Excessive levels of ROS can harm vascular endothelial cells and hinder the formation of new blood vessels, consequently slowing down bone repair [41]. OS affects angiogenesis in endothelial cells through several pathways. ROS mediate the lipopolysaccharide-induced breakdown of the adherens junction, resulting in increased endothelial barrier permeability and disrupted vascular homeostasis [42]. In addition, excess ROS can induce programmed death of endothelial cells, including pyroptosis, ferroptosis, and parthanatos [43].

### 3.2. Cartilage Callus Phase

The cartilage callus phase occurs at the site of chondrogenic osteogenesis. A soft callus, primarily composed of cartilage, forms around the fracture to stabilize the broken ends. This cartilage, although not part of the normal bone structure, serves as a temporary framework for the new bone to grow [44]. In contrast, intramembranous ossification directly generates new bone at the injury site without a cartilaginous intermediate.

Excessive ROS profoundly influence chondrogenesis within cartilage, impairing chondrocyte viability and differentiation, which are critical for cartilage formation during bone repair [45,46]. In the inflammatory state, ROS accumulate in chondrocytes and mitochondria are damaged [47,48]. ROS-induced oxidative stress leads to IκB-ζ stabilization and increases in IL-6 and MMP13 expression, both related to inflammation and cartilage degradation [49]. Furthermore, ROS not only directly compromise chondrocyte function, but may also interfere with essential signaling pathways such as BMP, thereby affecting cartilage maturation and mineralization, both of which are crucial for bone repair and regeneration [50,51].

### 3.3. Hard Bone Callus Phase

The cartilage callus phase is followed by the hard bone callus phase, which is characterized by the onset of mineralization. The soft cartilage callus is gradually replaced by a hard callus of bone in this phase [52,53]. Osteoblastic lineage cells, including osteoblasts and bone marrow mesenchymal stromal cells (BMSCs), play an important role in this process of bone formation. Osteoblastic lineage cells are particularly vulnerable to oxidative damage caused by ROS, which can diminish their viability and function, consequently reducing the rate of bone regeneration [54]. ROS can directly impair cellular components, including DNA, proteins, and lipids, hampering osteoblasts’ ability to proliferate and form new bone matrix [55]. OS has been shown to inhibit the Nrf2/HO-1, PI3K-Akt, and Wnt/β-catenin signaling pathways, which are critical regulators of osteoblast differentiation and bone formation [56]. This inhibition leads to decreased expression of osteogenic genes and diminished mineral deposition, both essential steps in the bone formation process. Moreover, high levels of ROS can induce apoptosis of osteoblasts [57,58]. This not only reduces the pool of cells available for new bone formation, but also skews the balance towards bone resorption, thus hindering the healing of bone defects.

### 3.4. Remodeling Phase

During the bone remodeling phase, the hard callus undergoes reshaping as newly formed bone tissue gradually approximates the original structure and strength of the bone. This process of fine remodeling depends on the coordination of osteoblasts and osteoclasts, in which osteoclasts are responsible for bone resorption and perform the same important function as osteoblasts [59]. Derived from hematopoietic precursors, osteoclasts dissolve old or damaged bone through the secretion of acidic substances and proteolytic enzymes, facilitating the removal of bone components [60,61,62]. Elevated ROS levels within a certain range play a pivotal role in the differentiation of osteoclasts, leading to an acceleration in bone resorption [63]. Specifically, ROS have been shown to activate the NF-κB and MAPK signaling pathways, two classical pathways that activate osteoclasts [64,65]. However, excessive ROS can also cause structural disorders of osteoclasts, dysfunction, and eventually apoptosis [63].

On the other hand, osteocytes, derived from osteoblasts, constitute the most abundant cell type in mature bone and play a pivotal role in sensing mechanical stress, thereby directing the remodeling process accordingly. They form an intricate lacunocanalicular network, facilitating the transport of nutrients and signaling molecules. However, when exposed to oxidative stress, the delicate balance of this finely tuned system can be disrupted. In addition to causing osteocyte apoptosis, the detrimental effects of OS on osteocytes are multifaceted [66]. Firstly, it can lead to a decrease in the production of sclerostin, a protein that osteocytes produce to inhibit bone formation by osteoblasts. This reduction can disrupt the normal regulatory feedback loop, potentially leading to an imbalance in bone remodeling [67,68,69]. Secondly, oxidative stress can augment the expression of RANKL by osteocytes, promoting osteoclast differentiation and bone resorption [70]. This further contributes to the imbalance between bone formation and resorption, hindering bone repair and reconstruction.

### 3.5. Effects of ROS Induced by Systemic Inflammation on Bone Repair

In specific pathological contexts such as diabetes, osteoporosis, infections, and aging, the detrimental effects of high levels of ROS on bone repair become more pronounced [71,72,73,74,75]. These conditions not only elevate ROS production, but also diminish the body’s capability to eliminate ROS, exacerbating oxidative stress. For instance, it has been shown that the hyperglycemic state of diabetes significantly increases the levels of ROS in the mitochondria and cytoplasm, affecting all stages of bone repair [76]. Therefore, in diabetic conditions, clearing excess ROS is critical to reducing oxidative stress, restoring stem cell mobilization, and promoting osteogenic differentiation [72]. Estrogen plays an important role in regulating redox balance by increasing the expression of antioxidant enzymes and restoring the overall antioxidant status, and when women are postmenopausal, ROS levels throughout the body increase significantly due to estrogen deficiency [77]. Therefore, for postmenopausal ROS increases, it is important to balance ROS levels by activating the expression of antioxidant enzymes. In addition, aging and long-term chronic inflammation can also lead to an increase in the overall levels of ROS in the body, thus affecting bone healing and repair [78,79,80].

Endogenous antioxidants such as antioxidant enzymes and non-enzymatic compounds play an essential role in bone homeostasis. Systemic diseases such as inflammation may increase ROS levels in the body by affecting the body’s endogenous antioxidant system, thus impeding bone repair activities. Jing Gao et al. found that SOD2 deficiency can cause mouse osteoblast dysfunction and significantly decrease bone mass by obstructing the clearance of excess mitochondrial superoxide and protein oxidation [81]. On the other hand, overexpression of mitochondrial catalase can effectively alleviate the ionizing radiation-induced reactive bone loss in mice [82]. Restriction of glutathione (GSH) synthesis, an important endogenous antioxidant, leads to acute degradation of RUNX2, impaired osteoblast differentiation, and reduced bone formation [83]. Therefore, when the endogenous antioxidant system is damaged during bone repair, it is necessary to supplement with exogenous antioxidants to promote new bone formation and bone healing.

### 3.6. Effects of NO (Nitric Oxide) and NO Synthase in Bone Repair

In the body, NO and NO synthase are important components of the oxidative stress system and play complex roles in the metabolic activities of bone tissue. As a free radical, NO is involved in the regulation of many physiological processes, such as bone metabolism, vasodilation, neurotransmission, and immune regulation [84,85,86]. Nitric oxide synthase (NOS), categorized into a neuronal form (nNOS), endothelial form (eNOS), and inducible form (iNOS), produces NO from molecular oxygen and guanine nitrogen (the terminal of the amino acid L-arginine). eNOS promotes osteogenic activity, but iNOS produced in large quantities in the inflammatory state inhibits osteogenic differentiation and promotes osteoclastic absorption [86,87,88,89]. It has also been reported that iNOS knockout mice had no change in bone mass [90]. In addition, nNOS knockout mice showed increased bone mass [91]. Albert Thomas Anastasio et al. reviewed the literature and discussed the delivery of NO through various biomaterials to promote fracture healing [92].

## 4. Classification of Antioxidants

Antioxidants can mitigate the harm of OS directly through reactions with free radicals or indirectly through inhibition of the activity of free radical-producing enzymes or improvement of the activity of intracellular antioxidant enzymes [93]. Common antioxidants include vitamins like C and E, polyphenolic compounds such as flavonoids and tannins, and various nanostructures that scavenge ROS. In the context of bone repair, antioxidants can be broadly categorized into naturally derived and synthetically engineered types. Naturally derived antioxidants predominantly consist of phytochemicals found in medicinal plants and dietary sources, while synthetic antioxidants are crafted in laboratories and customized to enhance specific therapeutic properties, such as increased bioavailability or targeted delivery to the site of bone damage.

### 4.1. Natural Antioxidants

Natural antioxidants play a crucial role in thwarting oxidation and impeding the growth of microorganisms [94]. They comprise a diverse array of compounds, such as flavonoids, polyphenols, natural pigments, vitamins, and antioxidant peptides.

Plants serve as abundant sources of exogenous antioxidants. Notably, fruits, vegetables, and select beverages like tea and wine are rich in flavonoids, polyphenols (e.g., epigallocatechin-3-gallate [EGCG], and resveratrol), and natural pigments (including anthocyanins and lycopene) [95,96]. These compounds possess the ability to counteract free radicals and modulate enzyme activity associated with OS. Moreover, well-recognized antioxidants like Vitamins C and E play pivotal roles. Ascorbic acid, or Vitamin C, directly scavenges free radicals, while vitamin E, a lipid-soluble antioxidant, shields cell membranes against oxidative harm [97,98].

Apart from the aforementioned exogenous antioxidants, there exist endogenous counterparts such as lipoic acid, uric acid, glutathione, and specific amino acids. For example, α-lipoic acid and its reduced form, dihydrolipoic acid, demonstrate direct free radical scavenging properties, and supplementation has been demonstrated to alleviate OS while replenishing diminished levels of other antioxidants [14,99]. Similarly, research has elucidated the pivotal role of GSH in maintaining cellular redox equilibrium. The thiol group within the cysteine amino acid is central to its function, acting as a potent reducing agent. This allows GSH to partake in a continuous cycle of reversible oxidation–reduction reactions, crucial for neutralizing ROS and preserving cellular health. Cells uphold a substantial concentration of the reduced form of glutathione through the assistance of glutathione reductase. Consequently, GSH can, in turn, reduce other enzymes and metabolites [100].

### 4.2. Synthetic Antioxidants

Synthetic antioxidants, engineered to replicate or even surpass the effects of natural antioxidants, have demonstrated significant potential in biomedical applications [101]. In 2007, Gao et al. initially reported on the intrinsic peroxidase activity of magnetic Fe_3_O_4_ NPs. These nanoparticles have garnered attention for their nanomaterial properties and enzyme-mimicking catalytic functions, and are termed Nanozymes [102]. Cerium oxide (CeO_2_ NPs) showcases remarkable catalytic prowess owing to oxygen vacancies within its lattice, facilitating redox cycling between Ce^3+^ and Ce^4+^ oxidation states [103]. This attribute enables CeO_2_ NPs to emulate diverse redox enzymes, including superoxide dismutase (SOD) and catalase, effectively scavenging ROS. Zinc-based antioxidants have also emerged as focal points of research, notably for their capacity to modulate cellular redox balance [104]. Zinc protects protein sulfhydryls or reduces the formation of free radicals by antagonizing the redox activity of metals such as iron and copper [105].

## 5. Administration of Antioxidants in Bone Repair

**Figure 3:**
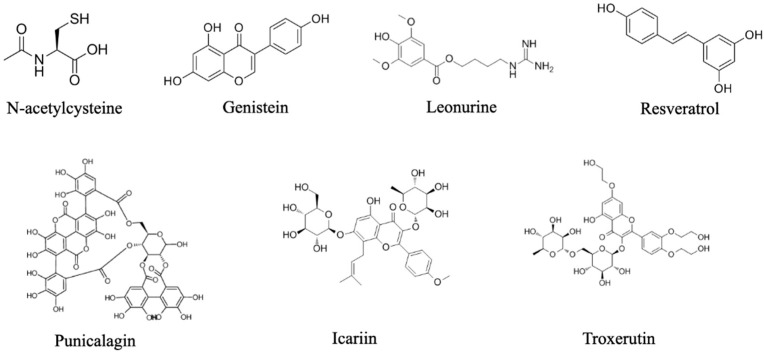
Chemical structures of representative antioxidants that have been reported in bone repair.

Most antioxidants, especially natural antioxidants, can be used directly in vivo to perform their function of clearing ROS and promoting bone repair (Figure 3 and Table 1).

For bone defects, topical drug treatment promotes bone healing and only applies to specific superficial sites. However, for repairing alveolar bone defects after tooth extraction, local drug administration proves highly suitable. Meng et al. demonstrated that topically applying N-acetylcysteine (NAC), a precursor for GSH, to alveolar defects in rats effectively reduces ROS levels, showing clinical potential for stem cell-based alveolar bone regeneration [106]. Nonetheless, some findings suggest that, while NAC may offer protective effects against OS, its impact on mesenchymal stem cell chondrogenesis and cartilage matrix development is complex and context-dependent [107]. Lei Huang et al. explored the administration of punicalagin, an active pomegranate polyphenol with antioxidant and anti-inflammatory properties, via daily local injections to treat bone defects at the condylar site of the femur in rats [108]. Their experimental results revealed that punicalagin effectively enhanced the osteogenic ability of BMSCs, promoted angiogenesis in endothelial cells, mitigated oxidative stress, and facilitated bone healing through the Nrf2/HO-1 pathway [108]. However, administering drugs locally through daily injections poses several inconveniences.

Oral ingestion or in vivo injection routes represent the most common methods of administering antioxidants for bone repair, allowing for the adjustment of dosing to regulate ROS levels in the body at various stages of bone healing. The physicochemical and pharmacokinetic properties of the drug determine whether to administer it orally or through injection. Genistein, a dietary polyphenol predominantly found in soy products, possesses antioxidant properties akin to quercetin, kaempferol, and resveratrol [109,110,111,112]. Govinda Bhattarai et al. employed intraperitoneal administration of genistein to shield gingival fibroblasts from LPS-induced stress, mitigating mitochondrial damage and ROS accumulation while preventing alveolar bone mass loss [109]. Similarly, Bingkun Zhao et al. utilized intraperitoneal injection of leonurine to activate mitochondrial autophagy, safeguarding BMSCs from oxidative stress and enhancing skull defect healing [113]. By subcutaneously injecting resveratrol, Govinda Bhattarai et al. curbed the production of inflammatory proteins, osteoclast formation, and circulating ROS in rats with periodontitis, effectively ameliorating alveolar bone loss [112]. Xiao Yang et al. stimulated osteogenic differentiation of stem cells and facilitated fracture healing through intramuscular injection of Troxerutin, a semi-synthetic derivative of the natural bioflavonoid rutin, renowned for its antioxidant effects [114]. In addition, icariin, an exogenous antioxidant extracted from the traditional Chinese medicine Herba Epimedii, has been reported to significantly reduce ROS levels in osteoblasts and inhibit iron-induced cell death via the NRF2/HO-1 signaling pathway when administered to rats via intragastric administration, thereby promoting osteoporotic fracture healing [115]. While oral methods are more convenient for clinical application compared to intraperitoneal injection, systemic administration affects the entire body. Therefore, the study of bone-targeted modification of antioxidant compounds is a very important research direction. For instance, Willson et al. utilized 3-hydroxy-4-pyrazole carboxylic acid as a carrier, coupled with diethylstilbestrol analogues, to develop compounds with bone-targeting ability. These compounds not only bind to hydroxyapatite in bone tissue, but also retain weak estrogen activity [116].

In addition to direct ROS scavenging, antioxidants play a significant role in modulating the inflammatory response associated with bone injury. Inflammation, while it is a natural part of the healing process, can exacerbate oxidative stress and impede recovery if left uncontrolled [62]. Antioxidants with anti-inflammatory properties, such as curcumin, offer further benefits to bone repair by downregulating pro-inflammatory cytokines and upregulating the Nrf2/ARE pathway, thus creating a more conducive microenvironment for bone tissue regeneration [117]. Moreover, studies have shown that curcumin enhances osteogenesis and cellular resilience against oxidative stress by modulating the Akt/Erk signaling pathways and preserving mitochondrial function in both rat BMSCs and human periodontal ligament stem cells [118,119]. These effects effectively reduce ROS levels, prevent hydrogen peroxide-induced damage, and promote cell survival, underscoring curcumin’s potential in regenerative bone therapies.

**Table 1:** Administration of antioxidant compounds in bone repair.

Antioxidant Compound	Application Method	Animal Model	Biological Effects	Ref.
N-acetylcysteine	Topical injection	Alveolar defects in rats	Upregulates PI3K/AKT signaling Enhances osteogenesis of dental follicle stem cells	[106]
Punicalagin	Topical injection	Femoral condyle defects in rats	Reduces oxidative stress via Nrf2/HO-1 pathway Enhances osteogenic differentiation in BMSCs and angiogenesis in HUVECs Promotes the polarization of M2 macrophages	[108]
Genistein	Intraperitoneal injection	Periodontitis bone defects in mice	Protects human gingival fibroblasts from LPS-mediated cellular ROS accumulation	[109]
Resveratrol	Subcutaneous injection	Periodontitis bone defects in rats	Inhibits the production of inflammatory proteins, osteoclast formation, and circulating ROS	[112]
Leonurine	Intraperitoneal injection	Skull defects in rats	Protects the proliferation and differentiation of BMSCs from oxidative stress by activating mitochondrial autophagy	[113]
Troxerutin	Intramuscular injection	Femur defects in rats	Increases the alkaline phosphatase activity, calcium nodule formation and osteogenic marker genes expression Activates Wnt/β-catenin signaling	[114]
Icariin	Intragastric administration	Fracture model in rats	Inhibits osteoblast ferroptosis via Nrf2/HO-1 signaling Promotes callus formation and enhances the transition from fibrous to osseous callus	[115]

## 6. Antioxidants Incorporated in Biomaterials for Bone Repair

**Figure 4:**
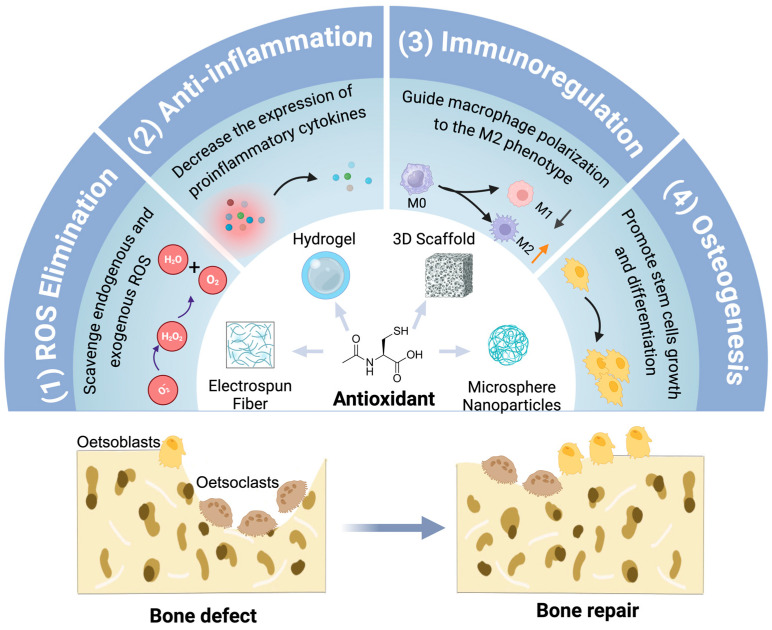
The effect of antioxidant-loaded biomaterial in bone repair. The combination of antioxidants and biomaterials can exert multi-targeted effects: (1) ROS scavenging; (2) anti-inflammation by decreasing the expression of proinflammatory cytokine; (3) immunoregulation by guiding macrophage polarization to the M2 phenotype; (4) osteogenesis by promoting the growth, proliferation, and differentiation of stem cells. The arrows in the figure indicate increasing (red) or decreasing (black). Created with BioRender.com (accessed on 17 June 2024).

In addition to direct administration for bone repair, antioxidant compounds have been extensively investigated in conjunction with biomaterials for the purpose of repairing bone defects. Commonly employed biomaterials include hydrogels, 3D scaffolds, microspheres/nanoparticles, electrospun fibers, etc. This integration can effectively enhance the healing of larger bone defect areas while also confining the effects of antioxidants to the specific site of the bone defect (Figure 4).

### 6.1. Hydrogels

Hydrogels, as injectable multifunctional scaffolds for bone regeneration, exhibit biodegradable properties and yield superior therapeutic outcomes when paired with antioxidants. This combination capitalizes on hydrogels’ inherent characteristics—such as biocompatibility, porous structure for cell infiltration, ability to mimic the extracellular matrix, and controlled release of bioactive compounds—alongside the antioxidative capabilities of various compounds to more effectively promote bone healing and tissue regeneration than either component alone [120]. Antioxidants embedded within hydrogels can scavenge ROS and modulate the inflammatory response, fostering an environment conducive to bone healing and regeneration.

Epigallocatechin-3-gallate (EGCG), when incorporated into hydrogels, scavenges ROS, bolsters cell survival, and fosters osteogenic differentiation under oxidative stress conditions [121]. An injectable thermosensitive hydrogel poly (D, L-lactide)-poly (ethylene glycol)- poly (D, L-lactide) system containing resveratrol and dexamethasone was injected and filled with irregular bone defect areas, creating a microenvironment conducive to osteogenesis, effectively scavenging excess free radicals at the damaged site by the powerful anti-inflammatory effects of resveratrol, and guiding macrophage polarization to the M2 phenotype [122]. Tannic acid embedded in hydrogels alleviates oxidative stress and guides macrophages toward an anti-inflammatory M2 phenotype, facilitating remodeling of implant-infected bone tissue [123]. Zhao et al. developed a self-assembled system incorporating Fucoidan, which aids in cartilage extracellular matrix (ECM) metabolism and ROS scavenging, fostering chondrocyte–ECM harmony through NRF2 pathway activation [124]. Moreover, hydrogels serve as a platform for the controlled and sustained release of antioxidants at the injury site, ensuring prolonged therapeutic effects. Ye et al. created a MnO_2_@Pol/HA hydrogel that delivers controlled antioxidant release and sustains ROS scavenging, preserving BMSC viability and osteogenic potential while inducing an anti-inflammatory shift in macrophage polarization, thereby effectively mitigating oxidative stress within the osteoporotic microenvironment [18]. In addition, the photosensitive hydrogel containing baicalin, a natural flavonoid with antioxidant capacity, designed by Li et al. can down-regulate the level of sclerosin in bone cells, promote osteogenic and vasogenic activities, and effectively promote the healing of bone defects of critical size in the skull [125]. Combining antioxidants with hydrogels can also alter the physical properties of the scaffold, including its stability and mechanical strength, making it suitable for bearer applications in bone tissue engineering. Wu et al. engineered the BPDAH-GPEGD hydrogel incorporating polydopamine/heparin nanoparticles, boasting enhanced mechanical properties with compressive strength exceeding 700 kPa and robust ROS scavenging capability, making it a promising scaffold for mandibular bone regeneration by addressing both structural integrity and oxidative stress [57].

In summary, hydrogels combined with antioxidants offer a multifaceted approach to bone repair, integrating enhanced osteogenesis, improved mechanical properties, and reduced oxidative stress and inflammation. This synergistic effect, akin to a “1 + 1 > 2” outcome, underscores the potential of these combinations in advancing bone tissue engineering and regeneration strategies.

### 6.2. 3D Scaffolds

Three-dimensional scaffolds infused with antioxidants create a structural and biochemical milieu conducive to bone regeneration, neutralizing ROS and fostering osteogenic differentiation while providing additional benefits. Qian et al. discovered that MnO_2_, integrated into a β-tricalcium phosphate and polycaprolactone scaffold, catalyzes the decomposition of hydrogen peroxide, thereby reducing local ROS levels and promoting osteogenic differentiation of BMSCs. This amalgamation can emulate the natural bone healing environment, furnishing a functionalized artificial periosteum [126]. Similarly, Wang et al. fabricated scaffolds via 3D printing technology, incorporating Reduced Glutathione Grafted Gelatine Methacrylate (GelMA-g-GSH). This scaffold activated the PI3K/Akt signaling pathway, augmenting cell proliferation and differentiation through the sustained release of reduced glutathione, thereby promoting osteogenesis under diabetic conditions [127].

Furthermore, the integration of metal ions such as zinc and cobalt within scaffold constructs confers additional advantages. Zinc-based scaffolds, modified with tannin, deliver antibacterial ions and support osteogenesis synergistically, addressing the multifaceted requirements for repairing infected bone defects [128]. Considering that inflammatory factors such as IL-1, IL-1β, IL-6, TNF-α, and ROS are widely expressed after cartilage degeneration and contribute to the inflammatory response, Wu et al. developed a hybrid scaffold using PLGA filled with a hyaluronic acid methacrylate hydrogel containing ROS-sensitive hyperbranched polymers with thioketal linkages to scavenge ROS, effectively promoting cartilage repair and regeneration [129]. In addition, Yuxiao Lai, Tianlin Liu, Xiaowei Xie et al. also constructed 3D scaffolds containing icariin, which can significantly promote bone defect repair [130,131,132]. This shows that icariin has good compatibility with a variety of biological materials. Tissue can also be an excellent scaffold material combined with antioxidants. Li et al. prepared small intestinal submucosal scaffolds combined with icariin, which significantly enhanced osteogenic activity and promoted the healing of skull defects in mice [133]. Similarly, collagen can be used as an excellent scaffold. Song et al. mixed duck paw collagen with quercetin and hydroxyapatite to prepare a scaffold, which promoted cell proliferation and osteogenic differentiation, as well as skull defect healing, in rats [134]. Different quercetin contents in scaffolds led to different biological effects. Song et al. found that low concentrations of quercetin in scaffolding can promote cell proliferation and osteogenic differentiation, while high concentrations of quercetin inhibit cell proliferation [135].

### 6.3. Electrospun Fibers

Electrospinning stands as a versatile and straightforward technique for fabricating ultrathin fibers, offering a fiber membrane with a high specific surface area that promotes cell adhesion and allows for the continuous and controlled delivery of drugs at local points [136,137]. Incorporating specific antioxidants into electrospun fibers can significantly accelerate the repair of bone defects and promote the range of bone healing.

Gao et al. devised polaprezinc-loaded polycaprolactone/gelatin hybrid electrospun nanofibers to craft a guided bone regeneration membrane with controlled polaprezinc release. This membrane exhibited antioxidant and osteogenic capabilities under both normal and oxidative stress conditions by upregulating Nrf2/HO-1/SOD1 signaling molecules in a concentration-dependent manner [138]. Lee et al. employed catechin, a plant flavonoid, for surface modification of polycaprolactone nanofiber membranes to enhance their biological function, creating a multifunctional matrix for repairing severe skull defects in mice [139]. Their study demonstrated efficient catechin coating on material surfaces, significantly increasing hydrophilicity and biocompatibility. This coating promoted adhesion, proliferation, and osteogenic differentiation of human adipose-derived stem cells, primarily through antioxidative and calcium-binding properties. Catechin-functionalized polymer nanofiber membranes notably enhanced in vivo bone formation in a critical-sized calvarial bone defect model [139]. Moreover, incorporating curcumin into electrospun fibers effectively inhibited critical factors like NF-κB and RANKL, pivotal in the inflammatory process and oxidative stress generation, thereby enhancing osteogenic differentiation [140]. The antioxidant icariin was added to electrospinning to achieve a continuously controlled release effect of icariin, which effectively promoted the skull defects in rats [141].

### 6.4. Microspheres/Nanoparticles

Due to their remarkable specific surface areas, nanosystems such as microspheres and nanoparticles enhance cell adhesion and proliferation, rendering them effective carriers for drug delivery systems [142,143]. The combination of antioxidant compounds with nanomaterials, leveraging their increased surface area, fully harnesses the functions of antioxidants, efficiently clearing ROS and promoting bone regeneration.

Moreover, nanosystems enable the precise delivery and controlled release of antioxidants at the injury site, ensuring sustained therapeutic effects. Wang et al. introduced injective programmable proanthocyanidin-coordinated zinc-based microsphere composite hydrogels for infected bone repair, emphasizing their ROS-responsive disintegration, antimicrobial activity, and support for osteogenesis [144]. Another study explored the synergistic antibacterial and anti-inflammatory effects of branched Au–Ag nanoparticles containing procyanidins for periodontitis treatment [145]. Qiu et al., by injecting tailor-made ROS-cleavable amphiphilic polymer nanoparticles coated with NAC into the periodontitis area, cleared ROS in the tissue, reduced inflammation, weakened osteoclasts, and promoted the regeneration of periodontal bone tissue [146]. Constructing an icariin delivery system by preparing micro/nano hybrid structured hydroxyapatite granules can significantly enhance osteogenesis and angiogenesis to repair bone defects [147]. In addition, Yuning Zhou et al. constructed hydroxyapatite bioceramic microspheres with a micro-nano hybrid surface containing quercetin, which could significantly heal femur defects of critical size in ovariectomized rats [148].

The presence of antioxidants within nanosystems modulates cellular pathways to favor bone healing, including promoting stem cell migration and differentiation and reducing inflammation. Nanosystems loaded with antioxidants overcome the limitations of traditional bone repair materials by offering higher surface area, promoting osteoblast function, and providing a conducive environment for bone tissue integration. The addition of antioxidants significantly enhances the bone repair activity of the microsphere/nanoparticle system, offering a new approach to bone defect repair applications with improved bioavailability of antioxidants.

The application of antioxidant-loaded biomaterials in bone repair are summarized in Table 2.

## 7. The Role of ROS/Antioxidants in Human Skeletal System

At present, although antioxidants are rarely used in the clinical treatment of human bone diseases, many antioxidants are being studied in clinical trials. In a randomized controlled clinical trial involving 44 patients, the researchers found that 1% melatonin gel effectively promoted periodontal bone tissue repair and significantly improved periodontal health [149]. This suggests that the application of antioxidants for the treatment of human bone defects is clinically promising.

More clinical research on the application of antioxidants in humans is being directed toward the prevention and treatment of osteoporosis in postmenopausal women. Lycopene has been reported to have the strongest antioxidant capacity among carotenoids, which upregulates RUNX2, ALP, and type 1 collagen and downregulates RANKL by activating the WNT/beta-catenin and ERK1/2 pathways [150,151]. In a study of 39 postmenopausal women, Russo et al. found that consuming tomato paste rich in lycopene significantly prevented bone loss [151]. In addition, Mackinnon et al. also found a significant reduction in the risk of osteoporosis in individuals using lycopene capsules through a 4-month clinical trial of 60 postmenopausal women [152]. Perilla seed oil is extracted from Perilla seeds. It is commonly used as vegetable oil and is rich in vitamin E, carotene and other antioxidant substances, with good antioxidant effects [153]. After a 1-year clinical trial, Kentaro Matsuzaki et al. found that long-term intake of perilla seed oil can improve age-related bone loss by inhibiting bone absorption and increasing α-linoleic acid levels [153]. Resveratrol, a natural polyphenol, was confirmed to be effective in increasing femoro-tibial density in postmenopausal women and reducing the risk of hip fracture within 10 years after a 24-month randomized, double-blind, placebo-controlled, two-phase crossover intervention trial [154]. In addition, several studies have shown that postmenopausal women taking antioxidants such as equol, ascorbic acid, alpha-tocopherol, vitamin E, and N-acetylcysteine can significantly relieve bone mass loss and promote the restoration of bone density [155,156,157,158,159,160]. These studies confirm the role of various antioxidants in promoting the metabolism of human bone formation, which provides important support for the application of antioxidants in the healing process of bone defects in humans.

## 8. Discussion

This article reviews the effects of ROS, a key substance in oxidative stress, on various stages of bone repair and describes the effects of various delivery methods of antioxidants on bone repair. It is worth noting that ROS play different roles in different stages of bone repair, and the complete elimination of ROS may negatively affect bone repair. Therefore, it is important to adjust ROS to appropriate levels at different times and in different spaces.

The key advantage of integrating antioxidant delivery systems into bone repair strategies lies in their local application, minimizing systemic side effects and enhancing efficacy in bone defect areas. These biomaterials, including nanoparticles, hydrogels, scaffolds, and electrospun fibers, have been meticulously designed to meet the complex requirements of bone healing, each with its own set of advantages and disadvantages. Nanoparticles and hydrogels are tailored for the controlled and continuous release of therapeutic drugs, ensuring precise delivery directly to the injury site. Conversely, stents and electrospun fibers not only serve as platforms for drug delivery, but also provide essential physical support for bone tissue engineering. Their structures promote the formation and vascularization of new bone, while their porosity enhances drug release kinetics [161,162,163]. Despite their considerable potential, these diverse biomaterials encounter several challenges. For instance, nanoparticles may present biocompatibility issues and long-term risks of adverse biological reactions. Additionally, effectively encapsulating therapeutic agents within nanoparticles poses a significant challenge, potentially limiting their therapeutic effectiveness [164]. The application of stents and electrospun fibers often necessitates surgical placement, which may heighten patient trauma and extend recovery periods. Moreover, while scaffolds offer robust support and strength, closely mirroring the natural hardness of bone and showcasing superior biomimetic and angiogenesis capabilities, scaffolds and electrospun fibers may encounter challenges in precisely controlling degradation rates and ensuring that mechanical properties are tailored to specific bone repair applications [165]. Furthermore, the requirements for ROS management vary at different stages of bone repair, imposing temporal and spatial demands on the ability of biomaterials incorporating antioxidant compounds to clear ROS effectively. Conversely, the direct application of antioxidant compounds, such as natural antioxidants, in bone defect repair offers convenience, such as through oral administration. However, their systemic effects lack targeting specificity. Therefore, the focus of subsequent research for the systemic application of antioxidants lies in bone-targeted modification. Numerous molecular groups with high affinities for bone minerals, such as rhein, bisphosphonates, and tetracyclines, have been identified in existing studies. Hybridizing these molecules with antioxidant compounds holds significant research potential [166,167].

The role of ROS in bone healing is two-sided, which increases the complexity of the strategy of regulating ROS to promote bone repair. While excessive ROS are recognized to impair bone regeneration by hindering osteoblastic differentiation and causing damage to cellular DNA, a controlled increase in ROS levels can yield beneficial effects, including antimicrobial activities crucial for treating infectious bone defects. Certain materials, such as Arginine Carbon Dots (Arg-CDs) and Zinc-Based Tannin-Modified Scaffolds, target bacteria at infection sites while simultaneously supporting bone regeneration [128,144,168]. These materials release ROS in response to the acidic environment of bone injuries, ensuring bacterial elimination without compromising osteogenesis. For instance, Arg-CDs not only eradicate bacteria through ROS, but also promote bone growth by enhancing osteogenic differentiation and macrophage polarization. These studies introduce a novel therapeutic strategy for treating bone defects by leveraging the dual action of ROS. This approach effectively regulates ROS concentrations in defective areas through antioxidant compounds, solves the problem of infection in bone healing, and promotes effective bone regeneration. This biomaterial underscores the precise regulation of the temporal and spatial distribution of ROS, marking a significant advancement in balancing antibacterial effects with tissue repair.

There are some potential conflicts with antioxidants in bone repair therapy strategies. Removal of ROS from the bone repair site may not be conducive to the removal of bacteria, which can lead to delayed bone healing and even exacerbation of infection. In addition, ROS at a physiological level contribute to cell signaling, whereas the use of antioxidants may inhibit normal cell signaling [169]. For example, Linrong Lu et al. found that feeding NAC increased the pathogenicity of T cell-induced autoimmune encephalomyelitis in mice, suggesting that antioxidant supplementation may carry the risk of promoting the onset and development of autoimmune diseases [170]. Thus, we should be cautious with the systemic use of antioxidants as well as the side effects of topical use. Although the clinical application of antioxidants for promoting bone healing has broad prospects, the mechanism of their systemic effects needs to be further investigated.

## 9. Conclusions

In this review, the research progress and mechanism of antioxidant compounds in bone defect repair were reviewed. In summary, delivery of antioxidant compounds to bone defect areas via targeted or localized drug delivery systems while precisely coordinating the spatial and temporal distribution of ROS is a promising therapeutic approach for bone defect repair. However, the success of these approaches depends on overcoming challenges related to the targeting and stability of drug delivery systems, as well as clarifying the complex role of ROS in bone regeneration. Future studies should prioritize the development of more stable and effective drug delivery platforms and strategies to precisely control ROS levels and optimize bone healing outcomes. In the application of antioxidants combined with biomaterials, more clinical trials are needed to support their safety and efficacy in human applications.

## Figures and Tables

**Table 2:** Application of antioxidant-loaded biomaterials in bone repair.

Biomaterials	Antioxidants	Animal Model	Biological Effects	Ref.
Hydrogel	Epigallocatechin-3-gallate (EGCG)	Bone defects in rabbits	Induce macrophages to polarize toward M2 phenotype Suppress inflammatory cytokines expression and improve osteogenesis related markers	[121]
Hydrogel	Resveratrol	Femoral defects in rats	Promote bone regeneration Guide macrophage polarization to the M2 phenotype Scavenge ROS	[122]
Hydrogel	Baicalin	Calvarial defects in rats	Promote osteogenesis and angiogenesis	[125]
Hydrogel	Tannic Acid	Implant-associated infection model in rats	Scavenging intracellular ROS Guide macrophages toward an anti-inflammatory M2 phenotype	[123]
Hydrogel	Fucoidan	Cartilage defects in rabbits	Promote extracellular matrix production and ROS elimination Promote neo-cartilage formation	[124]
Hydrogel	Hyaluronic acid	Segmental bone defect models	Eliminate ROS level in bone marrow and bone tissue Induce macrophages polarization from M1 to M2 phenotype Decrease the expression of pro-inflammatory cytokines	[18]
3D Scaffold	Glutathione	Calvarial defects in mice	Activate PI3K/Akt signaling pathway Augment osteoblasts proliferation and differentiation	[127]
3D Scaffold	Tannic acid	Femoral condyle defects in rats	Scavenge endogenous and exogenous ROS Control infection and promote osteogenesis	[128]
3D Scaffold	Icariin	Calvarial defects in mice	Enhance bone regeneration	[133]
3D Scaffold	Quercetin	Calvarial defect in rats	Increase cell proliferation Enhance bone regeneration	[134]
3D Scaffold	Icariin	Osteonecrosis of the femoral head in rabbits	Promote osteogenesis and angiogenesis	[130]
3D Scaffold	Icariin	Calvarial bone defects	Repair large-volume bone defects	[131]
3D Scaffold	Icariin	Steroid-associated osteonecrosis in rabbits	Facilitate bone regeneration Enhance the mechanical properties of new bone tissues Improve angiogenesis	[132]
3D Scaffold	Quercetin	Calvarial defects in rats	Promote cell growth, proliferation, and osteogenic differentiation Promote bone regeneration	[135]
Electrospun fiber	Polaprezinc	Calvarial bone defects	Protect proliferation and differentiation of MC3T3-E1 cells under oxidative stress Promote new bone formation	[138]
Electrospun fiber	Icariin	Calvarial defect in rats	Promote new bone formation	[141]
Electrospun fiber	Catechin	Calvarial defects in mice	Enhance proliferation, mineralization, and osteogenic differentiation of human adipose-derived stem cells (hADSCs) Promote bone formation by hADSCs transplantation	[139]
Electrospun fiber	Curcumin	Calvarial defects in mice	Inhibit NF-κB signaling and RANKL expression	[140]
Microsphere	Proanthocyanidin	Femoral condyle defects in rats	Promote M2 polarization of macrophages	[144]
Nanoparticle	Procyanidins	Periodontitis bone defects in rats	Inhibit inflammatory factors Regulate macrophage polarization	[145]
Nanoparticle	N-acetylcysteine	Periodontitis bone defects in rats	Increase expression levels of BMP2, RUNX2 and ALP Decrease osteoclast activity and local inflammation	[146]
Microsphere	Resveratrol	Femoral defects in rats	Regulate macrophage polarization to the M2 phenotype Promote osteogenic differentiation of mesenchymal stem cells	[122]
Micro/nano hybrid structured granules	Icariin	Femoral plug defects in rats	Promote new bone formation and blood vessel formation	[147]
Microsphere	Quercetin	Femur defects in rats	Promote osteogenesis and angiogenesis Inhibit osteoclastogenesis	[148]

## Data Availability

Not applicable.
